# Efficacy of Hyperosmolar Dextrose Injection for Osgood–Schlatter Disease: A Systematic Review with Meta-Analysis

**DOI:** 10.3390/diagnostics15101282

**Published:** 2025-05-19

**Authors:** Hye Chang Rhim, Lori B. Bjork, Jaehyung Shin, Jewel Park, Stephanie E. DeLuca, Katelyn C. McCarron, Ki-Mo Jang, Chris Ha

**Affiliations:** 1 Department of Physical Medicine and Rehabilitation, Harvard Medical School/Spaulding Rehabilitation, Boston, MA 02115, USA; 2 Department of Physical Medicine and Rehabilitation, Mayo Clinic, Rochester, MN 55905, USA; 3 Wallace H. Coulter Department of Biomedical Engineering, Georgia Institute of Technology, Atlanta, GA 30332, USA; 4 Department of Pediatrics, Los Angeles General Medical Center, University of Southern California, Los Angeles, CA 90033, USA; 5 Sports Medicine Service, Massachusetts General Hospital, Waltham, MA 02451, USA; 6 Department of Orthopaedic Surgery, Anam Hospital, Korea University College of Medicine, Seoul 02841, Republic of Korea; 7 Department of Physical Medicine and Rehabilitation, Mayo Clinic, Scottsdale, AZ 85259, USA

**Keywords:** Osgood–Schlatter disease, tibial tuberosity apophysitis, hyperosmolar dextrose injection, prolotherapy

## Abstract

**Background/Objectives:** Although Osgood–Schlatter disease (OSD) is often self-limiting following apophyseal closure, it may cause persistent symptoms into adulthood, affecting physical and functional activities. The purpose of this systematic review is to summarize the current evidence on the efficacy of hyperosmolar dextrose injection for patients with OSD unresponsive to conservative treatment. **Methods:** Multiple databases were searched for studies investigating the efficacy of hyperosmolar dextrose injection in patients with OSD. Two reviewers independently extracted data and evaluated the risk of bias. Meta-analyses were performed to compare hyperosmolar dextrose injection with placebo injections. **Results:** Four studies including three randomized controlled trials (RCTs) and one case series involving a total of 166 (162 males and 4 females) patients with 184 knees were included in this review. At three months, there was no significant difference in patient-reported improvement from baseline between hyperosmolar dextrose injection and placebo injections (standardized mean difference [SMD] = 1.92, 95% confidence interval [CI], −0.12 to 3.96; I^2^ = 96.2%). However, a meta-analysis of two RCTs including athletic pediatric patients found a pooled risk ratio of 2.11 (95% CI: 1.12 to 3.98, I^2^ = 30.73%) for pain-free return to sports at three months. In addition, at one year, a meta-analysis of two RCTs showed greater patient-reported improvement from baseline with hyperosmolar dextrose injection compared to placebo (SMD = 1.09, 95% CI, 0.62 to 1.56; I^2^= 0%). **Conclusions:** Based on the limited number of RCTs, although no improvement in patient-reported outcomes is seen at three months, hyperosmolar dextrose injection may safely facilitate a pain-free return to sports at three months and lead to patient-reported improvement at one year. However, further high-quality RCTs are needed to substantiate these findings.

## 1. Introduction

Osgood–Schlatter disease (OSD) is a common cause of anterior knee pain in adolescents, with an estimated incidence of 3.8 per 1000 person-years [1]. It predominantly affects active males more than females [2]. OSD is an apophysitis of the tibial tubercle caused by repetitive traction of the patellar tendon on the developing tibial tuberosity. This occurs particularly during periods of rapid femur growth, placing physically active adolescents undergoing rapid growth spurts at higher risk [3]. While OSD is typically self-limiting, a subset of patients may develop persistent pain, functional limitations, and swelling of the tibial tubercle [4].

Conventional management of OSD includes activity modification, physical therapy with a focus on strengthening and stretching the quadriceps, hip flexors, hamstrings, and pelvic stabilizers, patellar tendon straps, protective knee pads, oral and/or topical nonsteroidal anti-inflammatory drugs (NSAIDs), and cold therapy [4]. However, some patients do not respond to these conventional therapies. In one retrospective study, 26 out of 43 patients reported OSD-related knee pain during a median follow-up of 3.75 years, despite attempting strength exercises, stretches, activity modification, and oral medications [4]. Therefore, additional treatments, such as hyperosmolar dextrose injection, may be warranted for these patients.

Hyperosmolar dextrose injection, the most commonly used agent in prolotherapy, has been utilized for several decades to treat various musculoskeletal conditions [5]. It is hypothesized to work by creating an osmotic gradient that dehydrates and lyses local cells, leading to localized inflammation and initiation of the healing cascade. This inflammatory response stimulates cellular proliferation, extracellular matrix deposition, and collagen synthesis, promoting tissue repair and remodeling [6].

Although several randomized controlled trials (RCTs) [7,8,9] have evaluated the efficacy of hyperosmolar dextrose injections for OSD, the evidence is conflicting whether dextrose is superior to placebo. This systematic review with meta-analysis aims to summarize the current evidence on hyperosmolar dextrose injections for patients with OSD who did not respond to conservative management. We hypothesized that hyperosmolar dextrose injections may improve pain and facilitate a return to sports compared to placebo injections.

## 2. Materials and Methods

### 2.1. Study Design

PRISMA (Preferred Reporting Items for Systematic Review and Meta-Analyses) 2020 and PERSiST (implementing Prisma in Exercise, Rehabilitation, Sport medicine and SporTs science) guidance 2022 were used to conduct and report this review [10,11]. Our protocol was registered at the International Platform of Registered Systematic Review and Meta-analysis Protocols (INPLASY202520020). Our Population, Intervention, Comparison, and Outcome framework is as follows:

- Participants: Patients diagnosed with Osgood–Schlatter disease (tibial tuberosity apophysitis).
- Intervention: Hyperosmolar dextrose injection (prolotherapy) near/to the tibial tuberosity.
- Comparison: Placebo injection (saline or lidocaine), other injectable treatments, physical therapy, sham procedures, or no intervention.
- Outcome: Clinically relevant measures, such as Nirschl Pain Phase Scale (NPPS) and Victorian Institute of Sport Assessment (VISA), return to activity/sport, and adverse events.

### 2.2. Search Strategy

The literature was searched by a medical librarian for the concepts of OSD and dextrose or prolotherapy. Search strategies were created using a combination of keywords and standardized index terms. Searches were run on 6 February 2025 in ClinicalTrials.gov (2000+), Ovid Cochrane Central Register of Controlled Trials (1991+), Google Scholar through Harzing’s Publish or Perish for Windows, Ovid Embase (1974+), Ovid Medline (1946+ including epub ahead of print, in-process, and other non-indexed citations), Scopus (1788+), Web of Science Core Collection (Science Citation Index Expanded 1975+ and Emerging Sources Citation Index 2015+), and the World Health Organization’s ICTRP clinical trial registry (2005+). Full search strategies are provided in Supplemental Tables S1–S8.

### 2.3. Study Screening

All references were uploaded to Covidence and independently screened by two authors. Discrepancies between the two authors were resolved through discussion with a third author.

### 2.4. Study Selection

The systematic review aimed to include RCTs, prospective and retrospective comparative studies, and case series evaluating the efficacy of hyperosmolar dextrose injection for OSD. Reviews, case reports, studies conducted on animals, cadavers, or in vitro settings, letters to the editor, and technical descriptions were excluded. Studies lacking details on the intervention procedure, patient diagnosis, follow-up, clinical examination, or statistical analysis were excluded.

### 2.5. Data Extraction

Two authors independently reviewed each study identified in the initial search and conducted data extraction. Extracted variables included study design, inclusion and exclusion criteria, patient demographics, intervention details (dextrose concentration, volume, number of injections), rehabilitation protocols, follow-up durations, outcome measures, and adverse events. Any statistical information such as mean and standard deviation relevant for meta-analysis was also extracted.

### 2.6. Risk-of-Bias Assessment

All RCTs included in this review were assessed for the risk of bias using a revised Cochrane risk-of-bias tool for randomized trials. This tool assesses the risk of bias from five different domains, including the process of participant randomization, anomalies from interventions planned, absences of the outcome data, outcome measurements, and selective reporting of the result [12]. If all five domains were assessed to have a low risk of bias, then the overall risk of bias in the study was considered low. If up to two domains were assessed to have some concerns in risk of bias, then the overall risk of bias of the study was considered to have some concerns. Finally, if three or more domains were assessed to have some concerns in risk of bias or at least one domain was evaluated to have a high risk of bias, then the overall risk of bias of the study was determined to be high. Two authors assessed the risk of bias, and any discrepancies between the two authors were resolved through discussion with a third author.

### 2.7. Statistical Analysis

The standardized mean difference (SMD) was used to calculate the effect size because Topol et al. reported NPPS scores [7], while Nakase et al. and Wu et al. reported VISA scores [8,9], which represent two different patient-reported outcome measures. To provide a consistent interpretation of the effect size, the direction of NPPS scores was reversed (by multiplying by −1) [13], as lower NPPS scores indicate better outcomes, while higher VISA scores indicate better outcomes.

Wu et al. reported mean and standard errors for follow-up VISA scores instead of standard deviations (SDs), and therefore the SDs were calculated using the following formula [13]:

SD = SE × √n.

Meta-analysis was performed for outcomes at three months and at one year, as all studies reported three-month outcomes, and two studies reported one-year outcomes. A random effects meta-analysis was used to compare patient-reported improvement (measured by VISA or NPPS) from baseline to follow-up (three months or one year) in order to account for expected heterogeneity, including variability in patient populations and interventions. Because the included studies did not report within-group SDs for changes in scores, the following formula with an assumed correlation coefficient (r) of 0.5 was used [13,14]:

$$SD\_change=\surd ({SD\_baseline}^{2}+{SD\_post}^{2}-2r(SD\_baseline)(SD\_post))$$

Subgroup analysis was performed with two studies reporting VISA scores at three months, using a weighted mean difference (WMD). Furthermore, since the mean age of patients was significantly higher in the RCT by Wu et al. [8], we conducted a meta-analysis including only pediatric patients from the two RCTs [7,9]. From the same two RCTs, a meta-analysis of risk ratios (RRs) was conducted using the fixed-effects model in order to analyze the proportion of pain-free patients during sports at three months.

Statistical heterogeneity was assessed using Cochran’s Q test and I^2^ statistics, with I^2^ values categorized as low (≤25%), moderate (26–50%), or high (>50%) heterogeneity [15]. Publication bias was not assessed because less than 10 studies were included in the analysis. All analyses were conducted using STATA Version 16 (StataCorp, LLC, College Station, TX, USA).

## 3. Results

### 3.1. Study Selection

**Figure 1:**
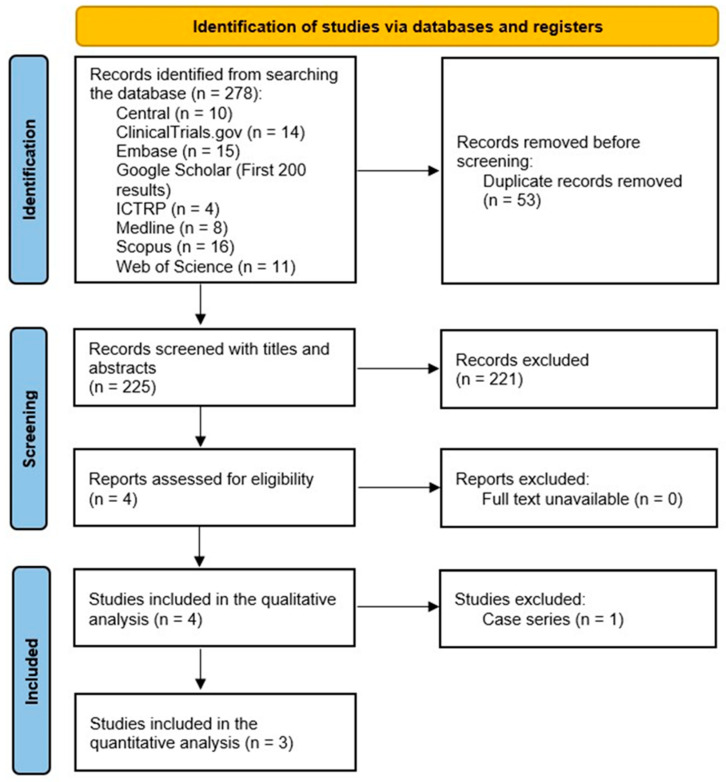
Preferred Reporting Items for Systematic Reviews and Meta-Analyses demonstrating the study selection process.

A total of 278 citations were retrieved. Deduplication was performed automatically in Covidence, leaving 225 citations for screening. After screening studies with titles and abstracts, 213 studies were removed, resulting in a total of 4 remaining studies, which all met the eligibility criteria (Figure 1). The four studies included in this review, including three RCTs [7,8,9] and one case series [16], had a total of 166 (162 males and 4 females) patients with 184 knees.

### 3.2. Study Characteristics

Population: Two RCTs [7,9] included exclusively pediatric patients (mean age 12–13 years) who participated in sports and were members of sports teams or clubs. One RCT [8] included male military officers and soldiers (mean age: 21 years). One cases series [16] included five pediatric patients (age range: 11–16) and one adult patient. The inclusion and exclusion criteria, along with demographic information, are summarized in Table 1 and Table 2.

Intervention: For hyperosmolar dextrose injection, the dextrose concentration ranged from 12.5% to 20%, with sterile water or lidocaine added to the injection mixture. Two studies [8,9] used ultrasound guidance, while the other studies [7,16] performed landmark-guided injections. Three RCTs [7,8,9] administered three injections monthly at 0, 1, and 2 months. The needle size ranged from 27 to 30 gauge. Details of the injectates and injection methods are summarized in Table 3.

Two RCTs [8,9] did not impose any activity restrictions, whereas one RCT [7] specified no running or kicking motions for one week following the first injection and for three days following the second and third injections.

Comparator: In two RCTs [8,9], lidocaine with saline injection was used as a placebo injection, while one RCT [7] included a placebo injection arm with lidocaine only and a usual care group undergoing supervised physical therapy.

Outcome: Two RCTs [8,9] evaluated VISA as their primary outcome measure, and one RCT [7] used the NPPS. Additionally, pain-free return to activities was also reported in two RCTs [7,9]. Follow-up durations varied from 1 month to 12 months. Two studies [7,16] did not report adverse events, whereas the other two studies explicitly stated that no adverse events occurred [8,9].

### 3.3. Risk-of-Bias Assessment

**Figure 2:**
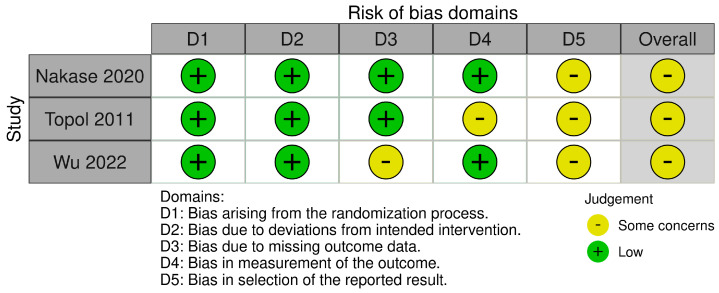
Risk-of-bias assessment for the included randomized controlled trials [7,8,9].

The RCTs included in this study were assessed to have some concerns about the risk of bias for their reported outcomes, including VISA, NPPS, and VISA-P (Figure 2) [7,8,9]. The primary reason for this assessment was due to the absence of clinical trial protocols important in the evaluation of selective reporting of the result domain. Therefore, all of the included studies were judged to have some concerns for risk of bias in this domain, resulting in their overall risk of bias being of at least some concern.

### 3.4. Meta-Analysis Results

A meta-analysis of three RCTs [7,8,9] demonstrated that at three months, there was no significant difference in patient-reported improvement from baseline between hyperosmolar dextrose injection and placebo injections (SMD = 1.92, 95% confidence interval [CI], −0.12 to 3.96; I^2^ =96.2%). However, at one year, a meta-analysis of two RCTs [7,8] showed greater patient-reported improvement from baseline with hyperosmolar dextrose injection compared to placebo (SMD = 1.09, 95% CI, 0.62 to 1.56; I^2^= 0%).

At three months, there was no significant difference in improvement from baseline between hyperosmolar dextrose injection and placebo injections in terms of VISA scores (WMD = 17.24, 95% CI −0.82 to 35.3; I^2^ = 90.1%) [8,9], or in pediatric patients (SMD = 0.819, 95% CI: −0.039 to1.676; I^2^ = 73.1%) [7,9].

Finally, a meta-analysis of two RCTs [7,9] including athletic pediatric patients found a pooled risk ratio of 2.11 (95% CI: 1.12 to 3.98, I^2^ = 30.73%) for pain-free return to sports at 3 months, indicating that patients receiving hyperosmolar injection therapy were 2.11 times more likely to become pain-free during sports at 3 months compared to the control group.

## 4. Discussion

Contrary to our hypothesis, there was no significant difference in patient-reported outcome improvement at three months between hyperosmolar dextrose and placebo injections. However, based on two RCTs, hyperosmolar dextrose injections may increase the likelihood of adolescent athletes with OSD returning to sports pain-free at three months and improving patient-reported outcomes at one year.

OSD is often described as a self-limiting condition with symptoms resolving in most patients upon apophyseal closure. Seldomly, symptoms can persist through adulthood, affecting physical or functional activities. Previous studies have reported that between 10% and 60% of patients may experience persistent symptoms into adulthood despite conventional management [17,18], although these figures should be interpreted with caution due to the small sample sizes in these studies. Nonetheless, athletes with a history of OSD have been found to exhibit higher levels of disability, as measured by the Knee Outcome Survey Activities of Daily Living Scale and the Sports Activity Scale [19]. These studies highlight the importance of adequate and early treatment of OSD at the time of initial diagnosis and the need for alternative interventions if conservative measures—such as activity modification, physical therapy (strengthening and stretching), and oral/topical medications—fail. Corticosteroid injections are commonly used for musculoskeletal conditions in adults [20], but they are not recommended in pediatric populations due to increased risks of potential growth plate injury [21] and tendon weakening or rupture [22,23]. Furthermore, there is a paucity of literature on treatments beyond conservative management, posing unique challenges in treating OSD patients with persistent symptoms.

The results of our study suggest that hyperosmolar dextrose injection may be a potential treatment option for young athletes who do not respond to initial conservative treatment but wish to return to sports, as well as for patients with persistent symptoms despite apophyseal closure. Hyperosmolar dextrose injection is a regenerative medicine therapy thought to stimulate local inflammatory responses and promote tissue healing through several proposed mechanisms. These include the increased production of platelet-derived growth factor (PDGF) [24], insulin growth factor-1 (IGF-1) [25], and transforming growth factor (TGF)-beta [26], which may lead to fibroblast proliferation and extracellular matrix deposition. While the exact mechanism of action is not fully understood, several systematic reviews have demonstrated potential benefits in various musculoskeletal conditions, including Achilles tendinopathy [27], plantar fasciitis [28], common extensor tendinopathy [29], rotator cuff tendinopathy [30], and osteoarthritis [31]. While our study is based on a limited number of trials, it contributes to the existing body of evidence supporting hyperosmolar injection therapy as a potential treatment option for patients with OSD.

### 4.1. Clinical Application

Given the limited number of studies included in the meta-analysis, the evidence is not robust enough to recommend hyperosmolar dextrose injections for all patients with OSD. Notably, analysis of our main outcome, patient-reported outcomes at three months, did not show any statistically significant difference between the hyperosmolar dextrose injections and placebo injections. Furthermore, our results should be interpreted with caution, as the potential benefits of hyperosmolar dextrose injections do not justify their routine use in these patients. However, based on our findings, it may be reasonable to consider this treatment for young athletes with persistent symptoms despite initial conservative measures, particularly those seeking additional options to facilitate their return to sports. Additionally, one of the included RCTs [8], which evaluated a slightly older population with apophyseal closure, highlights that symptoms of OSD may persist into adulthood and suggests that hyperosmolar dextrose injection therapy may be considered in this subset of the population.

Although only two studies in our review [8,9] utilized ultrasound guidance, ultrasound-guided injections are highly recommended to ensure precise delivery of the injectate, especially when it is delivered at multiple sites. In one RCT [9], injections were administered into the infrapatellar fat pad, deep infrapatellar bursa, and superficial infrapatellar bursa. In the other RCT [8], injections were placed in the superficial and deep layers of the patellar tendons. These anatomic targets may therefore be considered in clinical practice.

The included studies utilized 27–30-gauge needles, a factor that may be particularly relevant when treating pediatric patients, especially those who are averse to needles. Additionally, a potential advantage of hyperosmolar dextrose injections based on the reviewed studies is the lack of prolonged activity restrictions following injections. Two RCTs imposed no post-injection activity restrictions [8,9], while one RCT advised avoiding running and kicking for one week after the first injection and for two or three days after the second and third injections [7]. Most importantly, no studies reported any complications associated with the injections.

### 4.2. Future Research Direction

All three RCTs included in our review administered three injections at 0, 1, and 2 months. In his case series, Kidd reported that all but one patient responded well to just one or two injections [16], raising the possibility that fewer injections may still provide clinical benefit. Future studies should consider comparisons of different injection frequencies (e.g., single vs. multiple injections) and intervals to determine the most cost-effective and clinically beneficial dosing regimen. This is particularly important given that hyperosmolar dextrose injection in the United States is typically an out-of-pocket expense, which may be a barrier to access to this treatment.

Furthermore, while OSD is more prevalent in males, all three RCTs primarily included male patients. Future trials should consider more balanced sex representation or perform subgroup analyses to assess potential sex-specific differences in treatment response.

Lastly, standardized reporting of outcome measures, ultrasound guidance techniques, and injection formulations would enhance comparability across studies and facilitate evidence synthesis.

### 4.3. Limitations

The primary limitation of this meta-analysis is the small number of studies included. Therefore, caution is needed when interpreting the results of this review. A limited number of studies reduces statistical power and increases the likelihood that findings may be influenced by study-level variability, rather than true treatment effects. We also noted differences in patient populations, which may limit the generalizability of our findings to specific age groups. Wu et al. included adult military personnel [8], while the other RCTs focused on pediatric athletes [7,9]. However, we performed subgroup analyses to account for these demographic differences.

Variations in hyperosmolar dextrose injection formulations were also observed, with concentrations ranging from 12.5% to 20%, and some studies incorporated lidocaine or sterile water. Additionally, injection locations varied slightly across the studies, with two studies using landmark-guided injections. These methodological differences could contribute to clinical heterogeneity and may impact treatment efficacy. Given that patients may present with differing symptomatology and ultrasound findings, judicious clinical assessment remains essential to tailor the injection location, formulation, and volume to individual needs.

Lastly, all included RCTs were assessed as having some concerns regarding the risk of bias, mainly due to the lack of protocol registration or availability. The absence of a priori protocol registration may raise concerns about selective reporting, deviations from intended interventions, and incomplete reporting. To address these issues, future trials should register their protocols in advance, implement blinding when feasible, ensure adequate sample sizes, and report outcomes based on standardized guidelines.

These methodological limitations reduce the certainty of the evidence synthesized in this review, and therefore clinical applicability should be interpreted with caution until larger, well-designed, and transparently reported RCTs confirm these results.

## 5. Conclusions

Based on the limited number of RCTs, no significant difference in patient-reported outcome improvement was observed at three months between hyperosmolar dextrose and placebo injections. However, hyperosmolar dextrose injection may safely facilitate a pain-free return to sports at three months and lead to patient-reported improvement at one year. Further high-quality RCTs are needed to substantiate these findings.

## Figures and Tables

**Table 1:** Demographic characteristics of the included studies.

Study			Patient Demographics						
Author	Country	Study Design	Total	Male	Female	Group Characteristics	Mean Age (Years)	Mean BMI (kg/m^2^)	Symptom Duration (Months)
Nakase 2020 [9]	Japan	RCT	38 (43 knees)	37	1	Members of a sports club Prolotherapy: 22 knees Saline: 21 knees	Prolotherapy: 12.4 ± 0.9 Saline: 12.4 ± 1.2	NA	Prolotherapy: 6.7 ± 6.2 * Saline: 7.1 ± 8.2 *
Topol 2011 [7]	Argentina	RCT	54 (65 knees)	51	3	Members of an organized team involved in kicking or jumping sports Prolotherapy: 17 patients (21 knees) Lidocaine injection: 18 patients (22 knees) Usual care: 19 patients (22 knees)	Total: 13.3 [Range 10–17]	NA	Total: 8 ** [Range 3–72]
Wu 2022 [8]	China	RCT	70 (70 knees)	70	NA	Officers and soldiers in the military Prolotherapy: 35 patients Saline: 35 patients	Prolotherapy: 21.9 ± 4.8 Saline: 21.7 ± 4.4	Prolotherapy: 21.6 ± 1.9 Saline: 21.6 ± 1.8	NA

Abbreviations: BMI, body mass index; NA, not available; RCT, randomized controlled trial. * Mean symptom duration. ** Median symptom duration.

**Table 2:** Inclusion and exclusion criteria characteristics and main findings of the included studies.

Author	Country	Inclusion Criteria	Exclusion Criteria	Outcome Measures	Follow-Up Duration	Main Findings
Nakase 2020 [9]	Japan	Having anterior knee pain causing an inability to continue physical activities. Gradually worsening or acute symptoms following a trauma to the anterior tibial tuberosity directly. Having localized pain at the anterior tibial tuberosity area, which is aggravated by palpation, preventing eccentric and isometric knee extensions. History of conservative treatment not effectively working for more than a month.	Having patella instability, knee effusion, and proximal patella tendinopathy. Adults with OSD.	VISA	1, 2, and 3 mo	Significant improvements in VISA scores in both groups at 1 month, 2 months, and 3 months compared to baseline without any significant difference between groups. Complete resolution of pain during sports in seven knees (31.8%) from the prolotherapy group versus five knees (23.8%) from the control group at three months but not significantly different.
Topol 2011 [7]	Argentina	Having anterior knee pain and participating in kicking or jumping sports. No prior patellar origin tenderness or patellofemoral crepitus. Reproduction of pain at the tibial tuberosity while performing single-leg squat. Persisting pain for at least three months when playing sports. Tried progressive stretching of the hamstring, strengthening of the quadriceps, and progressive reintroduction to sports for at least two months. Informed consent received by the guardian and the patient.	Not reported	NPPS	3 and 12 mo	Significantly better improvement in NPPS for the dextrose injection group compared to the lidocaine injection group or the usual care group at three months. Significant improvement in NPPS for the lidocaine injection group than the usual care group. A total of 21 out of 21 patients in the dextrose injection group and 20 out of 22 patients in the lidocaine injection group had NPPS scores less than four, meaning unaltered sport, but 13 out of 22 patients in the usual care group had a score less than four, which indicated significant improvement for the dextrose and lidocaine injection groups compared to the usual care group. Significantly more patients had an NPPS score of zero at three months in the dextrose injection group than in the lidocaine injection and usual care groups. A similar trend was shown at 1-year follow-up with significantly more patients who were asymptomatic with sport in the dextrose injection group compared to the lidocaine injection or usual care group who did not change to receive dextrose injection.
Wu 2022 [8]	China	Having knee pain with ossification fragments in the patellar tendon insertion observed in MRI or X-ray and irregular ossification of the tibial tubercle. Served in the military for at least one year. History of discontinuing army training after undergoing conservative treatment for at least one month.	Having OSD on both sides of the knee or other conditions that could lead to pain in the knee. Finished active service in the past three months.	VISA-P	3, 6, and 12 mo	Significantly better VISA-P scores for the dextrose group than the saline group at all follow-up periods, 3, 6, and 12 months. Significantly improved VISA-P scores for dextrose and saline groups at 6- and 12-month follow-up periods following the first injection.

Abbreviations: mo, month; MRI, magnetic resonance imaging; NPPS, Nirschl Pain Phase Scale; OSD, Osgood–Schlatter disease; VISA, Victorian Institute of Sport Assessment; VISA-P, Victorian Institute of Sport Assessment—Patella.

**Table 3:** Treatment intervention characteristics of the included studies.

Author	Country	Intervention	Comparator	Injection Method	Post-Procedural Activity Restriction and Rehabilitation
Nakase 2020 [9]	Japan	1 mL 20% dextrose 1 mL 1% lidocaine	1 mL 1% lidocaine 1 mL saline	3 injections (0, 1, and 2 months) Ultrasound-guided 30-gauge needle First half of the solution into the infrapatellar fat pad and deep infrapatellar bursa Second half of the solution into the superficial infrapatellar bursa	No restrictions in sports activities
Topol 2011 [7]	Argentina	12.5% dextrose 1% lidocaine	Lidocaine injection: 1% lidocaine only Usual care: supervised physical therapy involving strengthening of the quadriceps and progressive stretching of the hamstring	3 injections (0, 1, and 2 months) Palpation-guided 27-gauge needle Needle inserted at the most distal point of the area of the pain or tenderness marked by palpation or doing a single-leg squat 0.5 mL then injected to the bone depth Injected 1 cm apart, translating proximally to the pain area One to two injections administered deep under the patellar tendon and targeted the tibia above the tuberosity Injected in any other pain areas detected by doing a single-leg squat until it became painless	Restricted running or kicking motion for one week following the first injection section and for three days following the second and third injections Could participate in sports with competition if the participants had shown a good result following the second injection Encouraged participants with NPPS scores less than three to play sports if they were painless Received illustrated sheets of exercises in strengthening quadriceps and progressive hamstring stretching
Wu 2022 [8]	China	12.5% dextrose solution (1 mL 50% dextrose, 1 mL sterile water, 2 mL 1% lidocaine)	Saline solution (2 mL 1% lidocaine, 2 mL saline)	3 injections (0, 1, and 2 months) Ultrasound-guided Total of 2 mL Pain area detected through palpation Injected 1 mL of the solution into each patella tendon’s superficial and deep layers	No restrictions on exercising or returning to usual work

Abbreviations: NPPS, Nirschl Pain Phase Scale.

## Data Availability

The data that support the findings of this study are available from H.C.R. upon reasonable request.

## Supplementary Materials

The following supporting information can be downloaded at https://www.mdpi.com/article/10.3390/diagnostics15101282/s1: Table S1: ClinicalTrials.gov; Table S2: Cochrane Central Register of Controlled Trials; Table S3: Embase; Table S4: Google Scholar; Table S5: International Clinical Trials Registry Platform; Table S6: MEDLINE; Table S7: Scopus; Table S8: Web of Science

## Abbreviations

The following abbreviations are used in this manuscript:

CI	Confidence interval
NPPS	Nirschl Pain Phase Scale
NSAID	Nonsteroidal anti-inflammatory drug
OSD	Osgood–Schlatter disease
SD	Standard deviation
SMD	Standardized mean difference
RCT	Randomized controlled trial
VISA	Victorian Institute of Sport Assessment
WMD	Weighted mean difference
