# Supplementary material for: Efficacy of Hyperosmolar Dextrose Injection for Osgood–Schlatter Disease: A Systematic Review with Meta-Analysis
Source: Diagnostics (Basel). 2025 May 19;15(10):1282. doi: 10.3390/diagnostics15101282 (PMC12110050; doi:10.3390/diagnostics15101282)
Supplement: Supplementary file 1 [file diagnostics-15-01282-s001.zip › diagnostics-3526384-supplementary.pdf]

Supplemental Tables

Supplemental Table S1. ClinicalTrials.gov (2000+)

Other terms: (OsgoodSchlatter OR Schlatter OR Lannelongue OR apophysitis) AND (dextrose OR glucose OR prolotherapy OR injection)

Supplemental Table S2. Cochrane Central Register of Controlled Trials (CCTR) via Ovid (1991+)

#	Query	Results from February 6, 2025
1	(osteochondr* or tibia* or shin or shinbone or knee* or OsgoodSchlatter * or Schlatter* or Lannelongue* or apophysitis).ab,hw,ti.	50,273
2	(OsgoodSchlatter * or Schlatter* or Lannelongue* or apophysitis).ab,hw,ti.	62
3	(dextro* or glucose or prolotherap* or ((proliferation or regenerat*) adj2 (therap* or inject* or treat*))).ab,hw,ti.	91,751
4	1 and 2 and 3	10

Supplemental Table S3. Embase via Ovid (1974+)

#	Query	Results from February 6, 2025
1	osteochondritis/ or osteochondrosis/ or exp tibia/ or (osteochondr* or tibia* or shin or shinbone or knee* or OsgoodSchlatter * or Schlatter* or Lannelongue* or apophysitis).ab,kf,ti,hw,dq.	446,093
2	(OsgoodSchlatter * or Schlatter* or Lannelongue* or apophysitis).ab,kf,ti,dq.	1,048
3	prolotherapy/ or glucose/	527,071
4	(dextro* or glucose or prolotherap* or ((proliferation or regenerat*) adj2 (therap* or inject* or treat*))).ab,kf,ti,dq,dy,tn.	1,143,305
5	or/3-4	1,143,438
6	1 and 2 and 5	15
7	from 6 keep 1-15	15

Supplemental Table S4. Google Scholar via Publish or Perish Version: 8.17.4863 (December 17, 2024)

First 200 Results:<br><br>(OsgoodSchlatter OR Schlatter OR Lannelongue OR apophysitis) AND (osteochondrosis OR osteochondritis OR tibia OR tibial OR shin OR shinbone OR knee) AND (dextrose OR glucose OR prolotherapy)

Supplemental Table S5. International Clinical Trials Registry Platform (ICTRP) from the World Health Organization (2005+)

standard interface:<br><br>(OsgoodSchlatter* OR Schlatter* OR Lannelongue* OR apophysitis) AND (dextro* OR glucose OR prolotherap* OR inject*)

Supplemental Table S6. MEDLINE via Ovid (1946+and Epub Ahead of Print, In-Process & Other Non-Indexed Citations and Ovid MEDLINE(R) Daily)

#	Query	Results from February 6, 2025
1	Osteochondrosis/ or Osteochondritis/ or Tibia/ or (osteochondr* or tibia* or shin or shinbone or knee* or OsgoodSchlatter * or Schlatter* or Lannelongue* or apophysitis).ab,kf,ti,hw.	342,067
2	(OsgoodSchlatter* or Schlatter* or Lannelongue* or apophysitis).ab,kf,ti.	783
3	or/1-2	342,067
4	Glucose/ or Prolotherapy/	181,935
5	(dextro* or glucose or prolotherap* or ((proliferation or regenerat*) adj2 (therap* or inject* or treat*))).ab,kf,ti,nm.	752,747
6	or/4-5	752,753
7	1 and 2 and 6	8

Supplemental Table S7. Scopus via Elsevier (1788+)

TITLE-ABS-KEY (osteochondr* OR tibia* OR shin OR shinbone OR knee* OR osgoodschlatter* OR schlatter* OR Lannelongue* OR apophysitis) AND TITLE-ABS-KEY (osgoodschlatter* OR schlatter* OR Lannelongue* OR apophysitis) AND (TITLE-ABS-KEY

(dextro* OR glucose OR prolotherap*) OR TITLE-ABS-KEY ((proliferation OR regenerat*) W/2 (therap* OR inject* OR treat*)))

Supplemental Table S8. Web of Science Core Collection via Clarivate Analytics (Science Citation Index Expanded 1975+ & Emerging Sources Citation Index 2015+)

#1	osteochondr* or tibia* or shin or shinbone or knee* or OsgoodSchlatter* or Schlatter* or Lannelongue* or apophysitis (Topic) and OsgoodSchlatter* or Schlatter* or Lannelongue* or apophysitis (Topic)
#2	dextro* or glucose or prolotherap* (Topic) or (proliferation or regenerat*) NEAR/2 (therap* or inject* or treat*) (Topic)
#3	#1 AND #2
